# Transcriptomic and Physiological Analyses Reveal the Molecular Mechanism through Which Exogenous Melatonin Increases Drought Stress Tolerance in Chrysanthemum

**DOI:** 10.3390/plants12071489

**Published:** 2023-03-29

**Authors:** Yan Luo, Taotao Hu, Yunyun Huo, Lingling Wang, Li Zhang, Rui Yan

**Affiliations:** 1 School of Agriculture, Ningxia University, Yinchuan 750021, China; luoyan1635752708@163.com (Y.L.); htt1961457327@163.com (T.H.); hyy1234561130@163.com (Y.H.); wanglingling0915@163.com (L.W.); zhang_li9988@163.com (L.Z.); 2 Ningxia Modern Facility Horticulture Engineering Technology Research Center, Yinchuan 750021, China

**Keywords:** *chrysanthemum*, exogenous melatonin, drought stress, transcriptome, plant hormone

## Abstract

*Chrysanthemum* (*Chrysanthemum morifolium* (Ramat.) Hemsl.) is an important species in China’s flower industry, and drought stress seriously affects the growth, quality, yield, and geographical distribution of this species. Melatonin (MT) plays a key role in regulating plant abiotic stress responses and stress resistance, but the mechanism through which exogenous MT regulates drought resistance in *chrysanthemum* remains unclear. This study explored the protective effect of MT on *chrysanthemum* drought tolerance and its key regulatory pathways. Exogenous MT application increased the photosynthetic capacity (Tr increased by 18.07%; Pn increased by 38.46%; and Gs increased by 26.52%) of *chrysanthemum* and attenuated decreases in its chlorophyll (19.89%) and relative water contents (26.94%). Moreover, MT increased the levels of osmolarity-related compounds such as soluble sugars (43.60%) and soluble protein (9.86%) under drought stress and increased antioxidant enzyme activity (SOD increased by 20.98%; POD increased by 35.04%; and CAT increased by 26.21%). Additionally, MT increased the endogenous MT (597.96%), growth hormone (45.31% and 92.09%), gibberellic acid (75.92% and 3.79%), salicylic acid (33.02%), and cytokinin contents (1400.00%) under drought stress while decreasing the abscisic acid (50.69% and 56.79%), jasmonate contents (62.57% and 28.31%), and ethylene contents (9.28%). RNA-seq analysis revealed 17,389, 1466, and 9359 differentially expressed genes (DEGs) under three treatments (PEG, MT, and MT _ PEG, respectively) compared with the control. Enrichment analyses of the DEGs identified more than 10 GO terms and 34 KEGG pathways. Nitrogen metabolism, sulfur metabolism, and alanine, aspartate, and glutamate metabolism were significantly increased under all three treatments. The DEGs included many transcription factors, such as *MYB*, *WRKY*, and *NAC* proteins. Our results preliminarily classify candidate genes and metabolic pathways with active roles in the interaction between MT and drought stress and advance the understanding of the molecular mechanism of the response to drought stress under MT conditions, thereby providing a theoretical basis for the breeding of drought-resistant *chrysanthemum*.

## 1. Introduction

Drought is one of the most serious abiotic stress factors affecting global crop yields [1]. Agriculture currently accounts for more than 70% of the world’s freshwater use (86% in developing countries), and this consumption is likely to increase as the global weather generally becomes drier and warmer [2]. Drought stress affects plant growth and development, photosynthesis, oxidative stress, water loss, osmotic regulation, hormone metabolism, and signal transduction to varying degrees [3]. Therefore, understanding the molecular mechanisms of the plant drought stress response and developing crops with enhanced drought resistance are crucial.

*Chrysanthemum* (*Chrysanthemum morifolium* (Ramat.) Hemsl.) is an important member of the family Asteraceae. A large number of C. morifolium cultivars are ornamental and medicinally important plants that are planted all over the world [4,5]. In addition to their esthetic value, some C. morifolium cultivars are used medicinally for their curative effects, particularly for treating headaches and the common cold [6]. As a species group in the *chrysanthemum* family, garden chrysanthemums are suitable for garden construction because of their low plants, dense flowering, long flowering period, and large crown size. *Chrysanthemums* began in 1961 when Chen Junyu cross-pollinated cultivated chrysanthemums with several wild and semi-wild chrysanthemums to select and breed a new group of varieties [7]. As an important worldwide horticultural crop, the *chrysanthemum* is strongly restricted by drought stress, which limits its ornamental quality and landscape applications [8]. Therefore, the development of *chrysanthemum* varieties with drought resistance is a current breeding goal [9]. The Arabidopsis *AtDREB1A* gene in *chrysanthemum* was previously shown to significantly enhance drought stress tolerance [10]. Additionally, the constitutive expression of *CdICE1* in the large-flowered cultivar ‘Jinba’ modulates *CgDREBa* expression, antioxidant enzyme activity, and proline contents to enhance the tolerance to drought stress [11]. Using overexpression and RNAi technology, the function of the *CmBBX24* gene in *chrysanthemum* and *Arabidopsis* was verified, and the results showed that this gene enhances freezing and drought tolerance [12]. The overexpression of *CmWRKY10* in the cut *chrysanthemum* cultivar ‘Shenma’ enhances the drought tolerance of transgenic plants [13]. The overexpression of *DgNAC1* in *chrysanthemum* can significantly increase its drought resistance compared with that of the wild type [14]. The heterologous overexpression of *CmBBX22* can delay leaf senescence and improve drought resistance in *Arabidopsis* [15]. The nuclear transcription factor *CmNF-YB8* is inhibited in *chrysanthemum* under drought stress conditions, and the tolerance of *CmNF-YB8*-silenced lines to drought stress was significantly enhanced, whereas the tolerance of overexpression lines to drought stress was significantly decreased [8]. Although we have identified some drought-resistance genes, there is little reference information available on the pathways of drought stress and the response of *chrysanthemum* to drought stress. Therefore, the exploration of potential plant growth regulators is of great significance for improving the resistance of *chrysanthemum* to drought stress.

Melatonin (MT), a ubiquitous hormone in animals and plants, can protect plants from a variety of biotic or abiotic stresses, including pathogen attack, drought, salinity, high temperature, low temperature, and heavy metal stress [16]. Studies have shown that MT is a powerful antioxidant in the plant response to abiotic stresses [17]. Under drought stress conditions, MT can scavenge large amounts of reactive oxygen species (ROS) generated in plants, enhance the activity of antioxidant enzyme systems, and control the extensive accumulation of hydrogen peroxide in plants, which results in the reduction of oxidative damage [18,19]. In rice [20], wheat [21], barley [22], cucumber [23], soybean [24], grape [25], alfalfa [26], and other plants, MT has been widely used to enhance resistance to a variety of stresses. Some studies have also indicated that MT improves stress resistance in *chrysanthemum* and that the exogenous spraying of MT can improve the resistance of *chrysanthemum* leaves to heat stress. However, the underlying molecular mechanism through which MT regulates the response to drought stress in *chrysanthemum* remains unknown.

In this study, to gain insight into the molecular mechanism of MT-mediated drought resistance, exogenous MT was applied to *chrysanthemum* seedlings, and their physiology and transcriptome were then comprehensively analyzed to explore the role of MT in the drought stress response. The results showed that MT improved the drought resistance of *chrysanthemum* at the physiological level by enhancing the photosynthetic capacity and the antioxidant defense system and reducing membrane damage and ROS accumulation. Furthermore, we focused on the analysis of key genes involved in starch and sucrose metabolism and hormone signaling pathways based on transcriptome and hormone data. This study provides comprehensive insights into the physiological and molecular regulatory mechanisms of MT-mediated drought stress tolerance in *chrysanthemum*.

## 2. Results

### 2.1. Effects of Melatonin on Chrysanthemum Seedlings under Drought Stress

**Figure 1:**
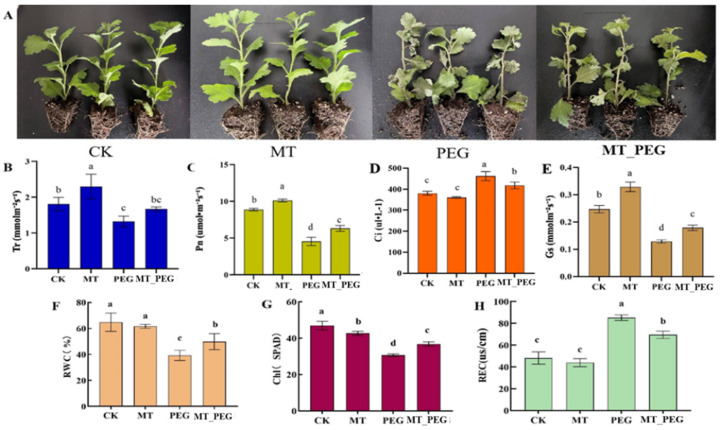
Effects of melatonin on *chrysanthemum* seedlings and their chlorophyll content and photosynthetic capacity under drought stress. (**A**) Phenotypes after 12 days of drought stress treatment. (**B**) Tr (Transpiration rate). (**C**) Pn (Net photosynthetic rate). (**D**) Ci (Intercellular carbon dioxide concentration); (**E**) Gs (Stomatal conductance). (**F**) RWC (Relative Water Content). (**G**) Chl (Chlorophyll content). (**H**) REC (Relative conductivity). CK, control; MT, exogenous melatonin treatment; PEG, drought stress; MT_PEG, drought stress with exogenous melatonin treatment. The data are presented as the means ± SEs (n = 3). Different letters indicate significant differences at *p* < 0.05.

Previous studies conducted in our laboratory have shown that the exogenous application of 100 μM MT could effectively alleviate the damage caused to *chrysanthemum* seedlings by drought stress [27]. Therefore, we treated *chrysanthemum* seedlings with 100 μM MT (MT), 20% PEG (PEG), or 100 μM MT and 20% PEG (MT_PEG), and water-treated seedlings were used as a reference for normal growth (CK). Different responses to drought stress were observed in the MT-treated and control seedlings (Figure 1A).

Photosynthetic gas exchange parameters can be used to measure the photosynthetic activity of *chrysanthemum* leaves. The effect of exogenous MT on the photosynthesis of *chrysanthemum* leaves is shown in Figure 1. The results showed that the photosynthetic parameters did not significantly differ between the CK and MT treatments. PEG stress significantly decreased the net photosynthetic rate (Pn), stomatal conductance (Gs), and transpiration rate (Tr) of *chrysanthemum* leaves and significantly increased the intercellular carbon dioxide concentration (Ci). The change in Gs tended to be the reverse of that in Ci, suggesting that nonstomatal limitations on Pn exist in plants treated with 100 μM MT. Additionally, MT-treated plants maintained a higher Tr and Pn, which was consistent with the higher Gs observed, suggesting that MT may enhance the plant root capacity for water uptake by taking advantage of plant growth. In conclusion, exogenous MT treatment slows the inhibitory effect of PEG on photosynthesis and maintains normal photosynthesis in *chrysanthemum* seedlings under PEG stress.

### 2.2. Effects of Melatonin on Oxidative Damage and Antioxidant Activity under Drought Stress

**Figure 2:**
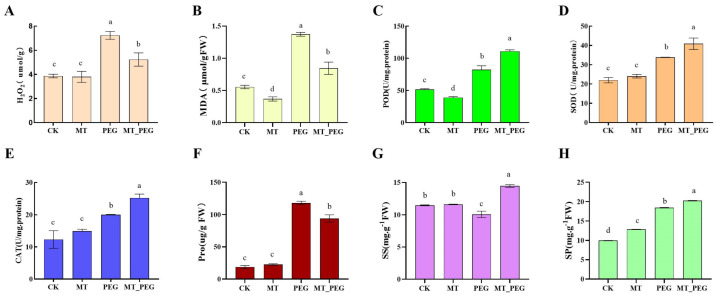
Physiological responses of *chrysanthemum* seedlings under drought stress. Contents of H_2_O_2_ (**A**) and MDA (**B**). Activities of POD (**C**), SOD (**D**), and CAT (**E**). Contents of proline (**F**), soluble sugar (SS) (**G**), and soluble protein (SP) (**H**). CK, control; MT, exogenous melatonin treatment; PEG, drought stress; MT_PEG, drought stress with exogenous melatonin treatment. The data are presented as the means ± SEs (n = 3). Different letters indicate significant differences at *p* < 0.05.

Drought stress disrupts the redox homeostasis of plants, which causes the accumulation of oxidant products. Thus, to examine the effects of MT on mitigating drought stress, the contents of oxidant products, including malondialdehyde (MDA) and hydrogen peroxide (H_2_O_2_), in *chrysanthemum* leaves were measured. The results showed that drought stress significantly increased the contents of H_2_O_2_ (Figure 2A) and MDA (Figure 2B) in *chrysanthemum* leaves. The exogenous application of MT significantly decreased the contents of H_2_O_2_ and MDA. These results indicate that MT can suppress the drought stress-induced production of oxidant products.

In order to explore the effect of MT on the antioxidant system under drought stress, the activities of the enzymes peroxidase (POD), superoxide dismutase (SOD), catalase (CAT), and proline (Pro), and the contents of soluble protein and soluble sugar were further investigated. The SOD, POD, and CAT contents in leaves treated with PEG+MT were significantly higher than those observed under PEG stress treatment (Figure 2C–E). Thus, the application of MT under drought stress improves antioxidant enzyme activity and increases the amount of antioxidant substances in the leaves of *chrysanthemum* seedlings, which results in improvements in their drought tolerance. Subsequently, the contents of proline, soluble sugar, and soluble protein were measured, and the results found that the contents under both drought stress and MT treatment were significantly higher than those of the control group (Figure 2F–H), indicating that the exogenous application of MT could improve the levels of organic osmotic adjustment substances in plants. The above results show that the application of exogenous MT under drought conditions can improve the antioxidant capacity of ground cover, reduce the content of endogenous ROS, and promote the growth of ground cover.

Proline, soluble sugar, and soluble protein can balance the water potential, increase the cytoplasmic osmotic pressure, and protect the membrane system, which results in reducing the water loss from cells. The results of this study showed that drought stress significantly increased the contents of proline (Figure 2F), soluble sugar (Figure 2G), and soluble protein (Figure 2H), resulting in biochemical changes in *chrysanthemum* seedlings. No significant change in the proline content was found in seedlings treated with MT alone. However, the proline content of *chrysanthemum* leaves treated with PEG_MT was significantly lower than that detected after the drought stress treatment. These results suggest that the soluble sugar, protein, and proline contents of *chrysanthemum* seedling leaves undergo different changes to facilitate the avoidance of drought stress, and the application of exogenous MT may reverse these changes.

### 2.3. Effects of Melatonin on the Synthesis of Endogenous Hormones and the Melatonin Content of Chrysanthemum under Drought Stress

**Figure 3:**
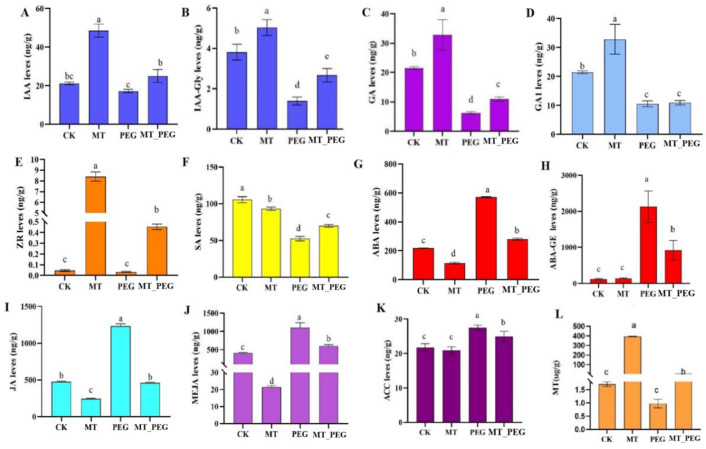
Effect of exogenous melatonin on the endogenous hormone levels of *chrysanthemum* seedlings under drought stress. Contents of (**A**) IAA (auxin), (**B**) IAA-Gly (Indole-3-acetyl glycine), (**C**) GA (Gibberellin), (**D**) GA1 (Gibberellin A1), (**E**) ZR (zeatin riboside), (**F**) SA (salicylic acid), (**G**) ABA (abscisic acid), (**H**) ABA-GE (abscisic acid-glucosyl ester), (**I**) JA (Jasmonic acid), and (**J**) MEJA (methyl jasmonate). Contents of (**K**) ACC (ethylene) and (**L**) MT (melatonin). CK, control; MT, exogenous melatonin treatment; PEG, drought stress; MT_PEG, drought stress with exogenous melatonin treatment. The data are presented as the means ± SEs (n = 3). Different letters indicate significant differences at *p* < 0.05.

The exogenous application of MT can combine with plant hormones to improve the stress resistance of crops, improve the quality of crops, and better regulate the growth and development of plants. To further study the effect of the interaction of MT with other plant hormones under drought stress, we detected the contents of endogenous IAA, ABA, JA, SA, GA, ZR, ACC, and MT in *chrysanthemum* leaves. The results showed that PEG treatment significantly reduced the contents of IAA (Figure 3A,B), GA (Figure 3C,D), ZR (Figure 3E), SA (Figure 3F), and MT (Figure 3L), and significantly increased the ABA (Figure 3G,H), JA (Figure 3I,J) and ACC (Figure 3K) contents in the leaves of *chrysanthemum* seedlings compared with those in the control group. Under drought stress, the external application of MT significantly reduced the contents of ABA, ACC, and JA in leaves. The above-mentioned results indicate that MT plays an important role in regulating the drought resistance of *chrysanthemum* and can effectively alleviate the damage to *chrysanthemum* seedlings caused by drought stress and suggest the existence of a certain relationship between the changes in endogenous hormone contents.

### 2.4. Transcriptome Analysis of Differential Gene Expression

**Figure 4:**
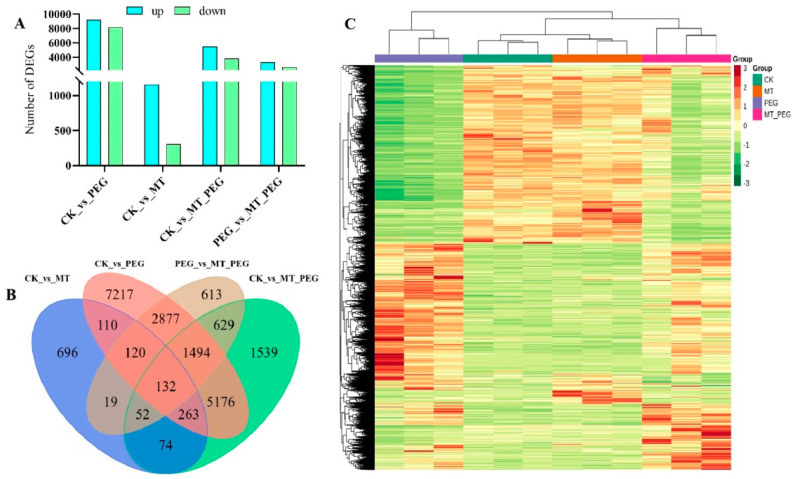
Overview of differentially expressed genes in *chrysanthemum* under different stresses. (**A**) DEGs (differentially expressed genes) in *chrysanthemum* under different stress conditions. (**B**) Venn diagram of DEGs from each comparison group. (**C**) Clustering heatmap of DEGs.

In order to reveal the effect of exogenous MT on gene transcription, *chrysanthemum* cuttings were subjected to the control (CK), drought stress (PEG), and MT_PEG treatments for 12 days and then to transcriptome sequencing. A total of 17,389 differentially expressed genes (DEGs) were identified between the control and drought treatment groups (CK vs. PEG), and these included 9201 up-regulated and 8188 down-regulated genes. A total of 1466 DEGs, including 1160 up-regulated genes and 306 down-regulated genes, were found between the control and MT treatment groups (CK vs. MT). A total of 9359 DEGs were identified between the control and MT_PEG treatment groups (CK vs. MT_PEG), and these included 5535 up-regulated and 3824 down-regulated genes. A total of 5936 DEGs were identified between the drought and MT_PEG treatment groups (PEG vs. MT_PEG), and these included 3297 up-regulated and 2639 down-regulated genes (Figure 4A). In addition, the DEGs among the four control groups were analyzed using a Venn diagram, and the results revealed 132 common DEGs (Figure 4B). A cluster analysis showed obvious separation between the samples, and repeated samples of each treatment were clustered together (Figure 4C).

### 2.5. GO Classification and KEGG Analyses

**Figure 5:**
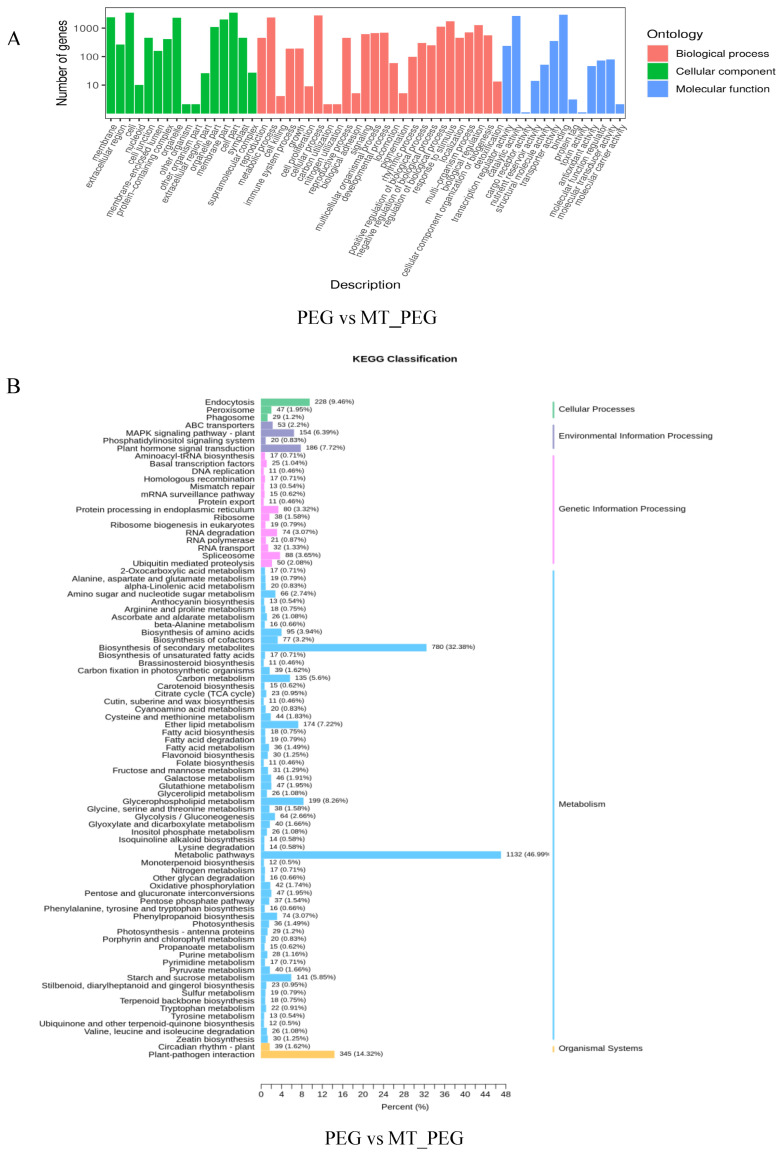
Functional annotation of DEGs identified from the various comparisons (PEG vs. MT_PEG) by GO and KEGG analyses. (**A**) GO analysis of DEGs identified from the comparison of the PEG vs. MT_PEG groups. (**B**) KEGG analysis of DEGs identified from the comparison of the PEG vs. MT_PEG groups.

GO classification and KEGG analyses were performed using transcriptome data on the effect of MT application on *chrysanthemum* seedlings under drought stress. For GO analysis, taking the drought treatment and MT_PEG treatment (PEG vs. MT_PEG) as an example, the DEGs identified from the comparison of the PEG vs. MT_PEG group were associated with 55 functional terms, including 26 biological process terms, 16 cellular component terms, and 13 molecular function terms. Among the identified biological processes, cellular processes and metabolic processes were related to the greatest number of genes. Among cellular components, cells, cell parts and organelles were the main terms associated with the DEGs. The analysis of molecular functions revealed that binding, catalytic activity, and transporter activity were the most enriched terms (Figure 5A and Supplementary Table S1).

The KEGG database is a comprehensive database that integrates information on genomes, biological pathways, diseases, and chemicals, among others. For KEGG analysis, taking the comparison of the drought and MT_PEG treatment groups (PEG vs. MT_PEG) as an example, the biological metabolic pathways were classified into five major categories: cellular processes, environmental information processing, genetic information processing, metabolism, and organismal systems. The largest number of genes were enriched in the metabolism (4340) category; among this category, the metabolic and biosynthesis of secondary metabolites pathways showed the greatest enrichment, with 1131 (46.99%) and 780 (32.38%) genes, respectively, and most genes exhibited up-regulated expression (Figure 5B).

### 2.6. KEGG Metabolic Pathway Analysis of DEGs

**Figure 6:**
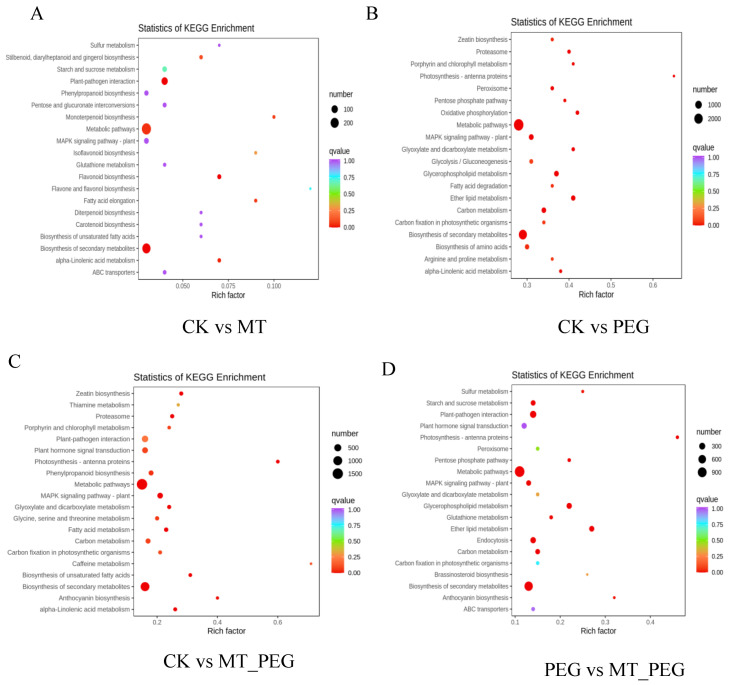
Enrichment scatter plot of KEGG pathways of DEGs identified from the comparisons of the four groups (CK vs. MT, CK vs. PEG, CK vs. MT_PEG, PEG vs. MT_PEG). The enrichment of KEGG pathways is shown in a scatter plot. The 20 most significantly enriched pathway entries are shown in this figure by default. (**A**) KEGG pathway enrichment scatter plot of the comparison of the CK vs. MT. (**B**) KEGG pathway enrichment scatter plot of the comparison of the CK vs. PEG groups. (**C**) KEGG pathway enrichment scatter plot of the comparison group of CK vs. MT_PEG groups. (**D**) KEGG pathway enrichment scatter plot of the comparison of the PEG vs. MT_PEG groups.

In order to confirm the main pathways through which MT contributes to drought tolerance, the DEGs were subjected to KEGG pathway enrichment analysis (Supplementary Table S2). In this analysis, a larger enrichment factor indicates greater enrichment, a larger spot indicates a greater number of DEGs enriched in the pathway, and the redder the spot is, the more significant the enrichment. As shown in Figure 6D, 2409 DEGs identified from the comparison of the drought and MT+PEG treatment groups (PEG vs. MT_PEG) were attributed to 135 pathways. The pathways containing more than 200 genes were metabolic pathways (1132), biosynthesis of secondary metabolites (780), plant–pathogen interaction (345), and endocytosis (228). The pathways associated with 150–200 genes were glycerophospholipid metabolism (199), ether lipid metabolism (174), *MAPK* signaling pathway-plant (154), and plant hormone signal transduction (186). Fewer than 150 DEGs were annotated to the other pathways. A total of 360 DEGs were found to be involved in plant signal transduction pathways, and plant hormone signal transduction (186), *MAPK* signaling pathway-plant (154), and phosphatidylinositol signaling system (20) were the most strongly related to drought stress signal transduction.

### 2.7. Effects of Exogenous MT on Transcription Factors in Chrysanthemum under Drought Stress

**Figure 7:**
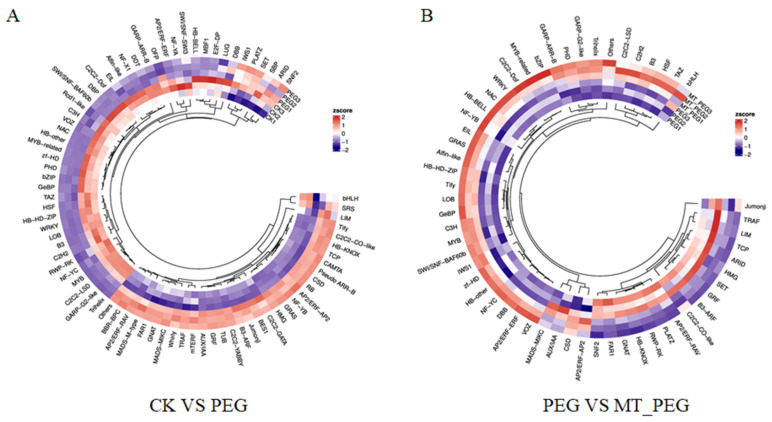
Heatmap of transcription factor families(CK vs. PEG and PEG vs. MT_PEG). (**A**) Transcription factor analysis in the CK vs. PEG comparison group. (**B**) Transcription factor analysis in the PEG vs. MT_PEG comparison group.

A total of 818 and 244 differentially expressed transcription factors (TFs) from 79 and 56 families were found from the CK vs. PEG and PEG vs. MT_PEG comparisons, respectively (Figure 7). Specifically, the *AP2/ERF-ERF* (APETALA2/ethylene-responsive element binding factor), *MYB* (myeloblastosis related), *WRKY*, *C2H2,* and *NAC* (NAM/ATAF1/CUC2) families responded to drought stress, and most of these were up-regulated. In addition, the *GRAS*, *AP2/ERF-ERF*, *MYB*, *NAC*, *Tify,* and *bHLH* (basic helix-loop-helix) families were strongly represented in the PEG vs. MT_PEG comparison, and most of these were down-regulated. Interestingly, the majority of genes in the *Tify* and *bHLH* families were down-regulated in the two comparisons, whereas the majority of genes in the *WRKY* family were up-regulated.

### 2.8. Validation of Sequencing Data by Quantitative Real-Time PCR (qPCR) Analysis

**Figure 8:**
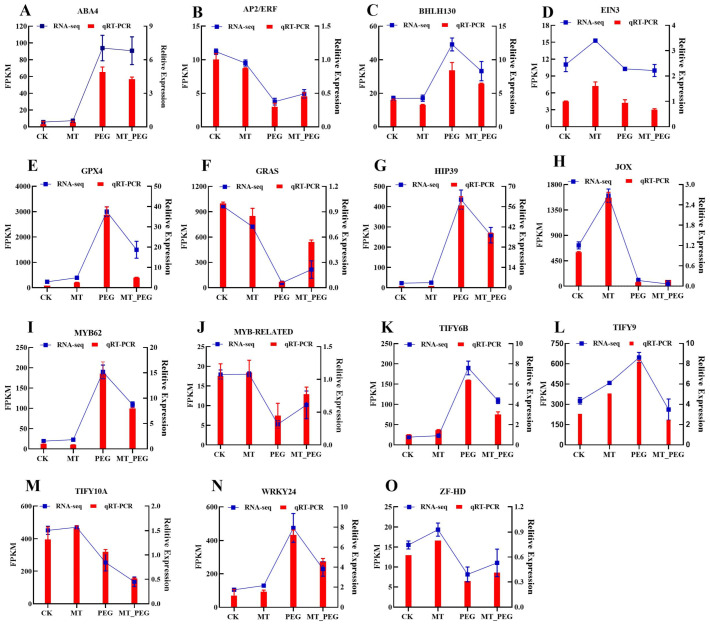
Accompanied by RNA-seq data, verification of phytohormones and transcription-factor-related DEGs by RT-qPCR. (**A**–**D**) Relative expression and RNA-seq data of genes related to *ABA4*, *AP2/ERF*, *BHLH130,* and *EIN3*. (**E**–**H**) Relative expression and RNA-seq data of two genes related to *GPX4*, *GRAS*, *HIP39,* and *JOX1*. (**I**–**L**) Relative expression and RNA-seq data of genes related to *MYB62*, *MYB-RELATED*, *TIFY6B,* and *TIFY9*. (**M**–**O**) Relative expression and RNA-seq data of genes related to *TIFY10A*, *WRKY24,* and *ZF-HD*. Error bars show the standard error between three biological replicates performed (n = 3).

In order to verify the quality of the transcriptome data, 15 genes were randomly selected for qRT–PCR analysis (Figure 8). The selected genes included phytohormone-related genes and genes related to key TFs associated with the response to adversity. The phytohormone-related genes include an Eth-related signaling pathway gene (*EIN3* and *AP2-ERF*), an *ABA-related* signaling pathway gene (*ABA4*), and a JA-related signaling pathway gene (*JOX*). The TF-related genes included *bHLH130*, three *TIFY-related* genes (*TIFY6B*, *TIFY9*, *TIFY10A*), a *WRKY* gene (*WRKY24*), and two *MYB* genes (*MYB62* and *MYB-related*). Other key genes involved in the stress response, such as *GRAS*, *HIP39*, and *ZF-HD*, were also identified. The expression trend found for the DEGs in the RNA-Seq analysis was basically consistent with that obtained by qRT-PCR. These results proved that the RNA-seq data were reliable.

## 3. Discussion

The observed damaging effects of drought stress on the studied parameters are well-known phenomena described in the published literature [28,29]. However, we identified an efficient effect of MT on enhancing plant growth under normal and stress conditions (Figure 1A). The significant improvement of these parameters due to the exogenous application of MT might be due to its active roles in various physiological processes or to its translocation to different plant parts, where it plays an important role in plant stress tolerance [30]. In this study, 100 µM exogenous MT was applied to *chrysanthemum* seedlings under drought conditions, and the plants were analyzed at the physiological and transcriptomic levels.

Different approaches are being used to reduce the marked effects of drought stress on plants, and these include the exogenous application of different chemicals/phytohormones through different methods [31]. A previous study showed that MT (N-acetyl-5-methoxytryptamine) is a multifunctional pleiotropic molecule with a wide range of cellular and physiological actions in living organisms, including animals and plants, that can effectively alleviate the plant redox imbalance induced by drought stress and prevent oxidative disturbance [32]. Additionally, an increase in antioxidant enzyme activities suggests that plants cope with drought stress by eliminating ROS [33]. Studies showed that melatonin-pretreated seedlings had higher antioxidant enzyme activities than untreated seedlings [34]. Likewise, it is generally believed that melatonin can increase the activity of antioxidant enzymes. In addition, melatonin can increase the efficiency of the mitochondrial electron transport chain, thereby easing electron leakage and reducing the generation of free radicals, which in turn protects antioxidant enzymes from oxidative damage [35]. Cui Guibin [36] found that the exogenous application of MT significantly increases the accumulation of antioxidant enzymes and enhances the synthesis of soluble sugars in PEG-stressed wheat seedlings. Zhang et al. [37] showed that the activities of SOD, POD, and CAT significantly increased 1.3-5.0-fold in MT-treated seeds under NaCl stress compared with untreated seeds. Ma et al. [38] found that the exogenous application of 500 μM MT significantly increases the activities of SOD, CAT, and APX in cassava (*Manihot esculenta* Crantz) roots. The results of this study showed that MT treatment significantly increased the H_2_O_2_, SOD, POD, and CAT activities and significantly decreased the MDA and Pro contents of *chrysanthemum* seedlings under drought stress compared with those observed without MT treatment. These findings indicate that MT treatment increases antioxidant enzyme activities and the accumulation of soluble sugars in *chrysanthemum* seedlings under drought stress and slows the peroxidative and osmotic damage caused by drought stress.

Some studies have shown that drought stress inhibits plant growth, and exogenous MT can alleviate drought-induced growth inhibition [39]. There are also studies that show under high salt conditions, seed germination and root elongation were inhibited, plant growth was decreased, and net photosynthetic rate and chlorophyll content were decreased, whereas pretreatment with exogenous melatonin allowed plants to maintain robust roots, reduced growth inhibition, and improved photosynthetic capacity [40]. Recently, similar data have been obtained in salt-stressed Bermuda grass, citrus, and sunflower, which confirmed the protective role of melatonin on photosynthetic pigments [41]. This conclusion was confirmed by the results of the present study. The analysis of photosynthetic plasticity under drought-stress conditions is an effective method for determining the water sensitivity of horticultural crops [42]. Our data showed that the Pn decreased when the Gs were reduced by drought stress. Pretreatment with MT led to greater Gs and a significant increase in the photosynthetic capacity because the stomata were more likely to remain open. According to a previous study [43], stomatal limitation of the Pn occurs only when the Gs decreases in parallel with a decline in the Ci. In this study, the change in the Gs notably tended to show the opposite pattern of that in the Ci, suggesting that nonstomatal limitations of the Pn exist in plants administered 100 μM MT, whereas the 100 μM MT_PEG groups showed higher efficiency of CO_2_ conversion. Furthermore, MT-treated plants maintained a higher Tr, which was consistent with the higher Gs, suggesting that MT may enhance the plant root capacity for water uptake. In a study of maize, Zhao Chengfeng [44] found that exogenous MT-treated plants exhibited a higher Pn and Gs and a lower intercellular Ci under drought stress than drought-stressed plants that were not treated with MT, indicating that exogenous MT exerts a mitigating effect on photoinhibition under drought stress. Therefore, MT spraying is an important method for enhancing the photosynthetic adaptation of *chrysanthemum* seedling leaves to a dry environment.

Previous studies have described various tolerance mechanisms based on the physiological changes that overcome the deleterious effects of drought [45]. Alterations in the endogenous plant hormone levels are one of the most important (Figure 3). As crucial signaling molecules, plant hormones resist or adapt to drought stress by jointly responding to water deficits under drought stress [46]. Some studies have shown that adversity stress affects the content of some plant hormones, such as GA and ABA, inhibiting plant growth [47,48,49]. For example, the exogenous application of melatonin can increase the salt tolerance of cucumber by mediating the expression of the genes associated with the biosynthesis and catabolism of GA and ABA [37]. Under drought stress conditions, IAA and GA play a crucial role in horticultural plant growth [50]. In the present study, drought stress decreased the IAA, GA, ZR, SA, and endogenous MT contents in *chrysanthemum* leaves and promoted an increase in the ABA, JA, and ACC contents, while the opposite results were obtained after exogenous MT application under drought stress conditions, indicating that the MT spraying of plants under drought stress helps increase the endogenous hormone levels, promote plant growth and improve plant resistance. The effect of drought stress on IAA is highly complex and mainly manifests as large differences between organs and growth periods of plants [51]. Some studies have concluded that drought stress leads to a significant increase in the ABA content and that a higher degree of drought is associated with a greater ABA concentration [52,53]. This finding is consistent with the results of this study. However, certain studies have suggested that stomatal closure and a decrease in the transpiration rate are the results of the combined action of ABA and ZA [54]. The high accumulation of SA in plants under drought stress protects them from oxidative stress and increases their tolerance to drought stress [55]. Thus, the above results indicate that interactions among various hormones under drought stress confer tolerance and resistance to plant species [31].

Previous similar studies have shown that drought stress can alter the response of *chrysanthemum* plants at morphological, physiological, and molecular levels [56]. In order to compare DEGs among multiple treatment groups under drought stress, four pairwise comparisons were performed: CK vs. MT, CK vs. PEG, CK vs. MT_PEG, and PEG vs. MT_PEG. The numbers of DEGs and Venn diagram analysis results are shown in Figure 4. The CK vs. PEG comparison yielded the largest number of DEGs, followed by the CK vs. MT_PEG and CK vs. MT comparisons. These results suggested that under drought stress, MT treatment increased the numbers of both total DEGs and down-regulated DEGs. Similarly, Debnath et al. [57] showed exogenous MT application protected tomato plants from stress by altering the expression patterns of DEGs, particularly secondary metabolite-and TF-related genes. Notably, all these transcriptomes identified a large number of transcription factors as DEGs; the functional identification of these DEGs may provide more valuable clues into melatonin-mediated signaling.

TFs are critical regulators of plant development and essential plant activities such as responses to environmental stresses and hormones [58]. Previous studies have identified several genes encoding TFs, including *DREB/CBF*, *WRKY*, and *MYB* TFs, involved in MT-mediated stress signaling pathways in plants [41,59]. In this study, the expression levels of a set of TF genes, including *AP2/ERF*, *ABA4*, *TIFY,* and *WRKY* genes, were found to be significantly up-regulated in drought-stressed seedlings. WRKY proteins are important regulators of various physiological processes, including pathogen defense, senescence, and trichome development [60]. Studies with similar results to this one show that the induction of melatonin increases the transcripts of some stress-related transcription factors and the underlying downstream genes, activates *MAPK* signaling, and up-regulates carbohydrate metabolism, especially of sugars [61]. Based on these results, the MT-mediated up-regulated expression of the genes encoding these TFs in *chrysanthemum* seedlings under drought stress suggested that the drought-induced inhibition of growth and development was offset.

## 4. Material and Methods

### 4.1. Plant Materials and Treatments

Garden chrysanthemum cuttings were purchased from Beijing Flower and Wood Company. MT was purchased from Solarbio. Equal-stemmed cuttings with a length of 7 cm were prepared from healthy garden chrysanthemum morifolium ‘Xuanqiu ninghong’ plants. The cuttings were placed in a perforated tray with a mixed substrate (peat: coconut bran: perlite = 2:3:2) and allowed to root for 2 months. Thereafter, the chrysanthemums were subjected to four treatments: (a) well-watering without any other treatment (CK); (b) 100 µM MT root irrigation (MT); (c) 20% polyethylene glycol (PEG) root irrigation; and (d) 100 µM MT foliar spraying plus drought stress root irrigation (MT_PEG). The plants were treated with MT by foliar spraying for 3 days (once a night) before 20% PEG root irrigation to impose drought stress. The other three treatments were performed by administering 300–350 mL of water, 100 µM MT, or 20% PEG every three days for 12 days. Fertilizer was applied by root irrigation once a week at an N:P:K ratio of =20:20:20, 800 times per gram to water. All experiments were conducted in a greenhouse at the Ningxia Horticulture Industrial Park, China. The greenhouse was maintained with a 15/9 h (day/night) photoperiod, the average temperature was 25/20 °C (day/night), and the relative humidity was 65%. Importantly, the optimal concentrations of MT and PEG were selected based on preliminary experimental results. The experiments were conducted in triplicate using pooled samples. Sampling and index determination were performed after the experimental treatments. Freshly harvested leaves were rapidly frozen in liquid nitrogen and stored at −80 °C.

### 4.2. Determination of Physiological Indicators such as Light and Plant Characteristics and Chlorophyll and Antioxidant System Contents

Physiological measurements: The chlorophyll content was measured using a portable Soil Plant Analysis Development (SPAD) chlorophyll meter. The relative water concentration (RWC) was measured according to a previously described method [62]. The relative electrical conductivity (REC), superoxide dismutase (SOD) activity, and peroxidase (POD) activity were determined according to the method described by Gao Junfeng [63]. Malondialdehyde (MDA) was measured using the method described by Zhao Shijie [64]. The activities of hydrogen peroxide (H_2_O_2_) and proline (Pro) were analyzed using a Solarbio kit (Beijing, China), following the manufacturer’s instructions. Catalase (CAT) activity was determined according to the method described by Li Hesheng [65]. The soluble sugar and soluble protein contents were determined by anthrone colorimetry [66] and staining with Coomassie brilliant blue G-250 [67].

Photosynthetic parameters: The Pn, Tr, Ci, and Gs were measured using a Li-6800 portable photosynthesis system (LI-COR, Lincoln, NE, USA). Measurements were taken between 9:30 am and 11:00 am on the day after treatment, and three plants were randomly selected from each treatment.

### 4.3. Determination of Endogenous Hormone and Melatonin Contents

In this study, a Qtrap 6500 mass spectrometer was used to quantitatively analyze the endogenous hormones in *chrysanthemum* leaves by the ESI-HPLC-MS/MS method. The hormones included IAA, ABA, JA, GA, SA, ZR, ACC, and MT.

### 4.4. Transcriptome Sequencing and RNA-Seq Data Analysis

Transcriptome sequencing data were provided by MetWare Biotechnology (Wuhan, China). For analysis of the RNA integrity and the presence of DNA contaminants by agarose gel electrophoresis, the purity of total RNA was measured with a Qubit 2.0 Fluorometer (Life Technologies, Santa Clara, CA, USA), and the RNA integrity was assessed using an Agilent Bioanalyzer 2100 system (Agilent Technologies, Santa Clara, CA, USA). High-quality total RNA samples were reverse transcribed into cDNA and used for cDNA library construction. After library inspection, the different libraries were pooled according to the target downstream data volume and sequenced using the Illumina platform. After sequencing, clean reads were obtained by removing reads containing adaptors, paired reads with sequences with more than 10% unknown nucleotides (N), and reads with a quality rating of less than 50% (Q-value ≤ 20). Sequence alignment with the specified reference genome, differential expression analysis, functional annotation of DEGs, functional enrichment based on the expression of genes in different samples or different sample groups, and other expression-level analyses were performed.

Differential expression analysis between sample groups was performed using DESeq2 [68,69] to obtain DEG sets between two biological conditions; the differential analysis requires unstandardized read count data of input genes and not standardized data such as RPKM and FPKM. After the differential analysis, the false discovery rate (FDR) was obtained by correcting the probability of hypothesis testing for multiple hypotheses using the Benjamini–Hochberg method. The screening conditions for DEGs were |log_2_Fold Change| ≥ 1 and FDR < 0.05. To improve the accuracy of the DEGs, genes with a fold change greater than 2 and Q-value ≤ 0.001 were defined as significant DEGs for DEGs identified in multiple comparisons. For GO enrichment, the DEGs were classified into several functional groups according to GO annotation results and the official classification method. The Phyper function in R software was used for the enrichment analysis, the p value was calculated, and an FDR correction was performed. A GO function with a corrected *p* value ≤ 0.05 was considered significantly enriched. According to the KEGG pathway annotation classification, the Phyper function in R software was used for enrichment analysis, and the *p* value was calculated and then corrected by FDR. Similarly, a function with Q-value ≤ 0.05 was regarded as significantly enriched.

### 4.5. Quantitative Real-Time PCR (qPCR) Analysis

Fifteen DEGs were randomly selected, primers were designed using Primer Premier 5.0, and the same samples were used for qRT-PCR to verify the RNA-seq data quality(Supplementary Table S3). Reverse transcription was performed using the Hifair^®^ III 1st Strand cDNA Synthesis SuperMix for qPCR (gDNA digester plus) first-strand synthesis kit (11141es60). qRT-PCR analysis was performed using the Hieff^®^ qPCR SYBR Green Master Mix (Low Rox Plus) kit. The program was as follows: an initial step of 95 °C for 30 s, 40 cycles of 95 °C for 30 s, 60 °C for 10 s, and 72 °C for 30 s, and melting curve analysis. Quantitative results were calculated using the 2^-ΔΔCT^ method [70,71]. Three technical replicates and three biological replicates of each group were included in the experiments. In this study, all qRT-PCR was performed with EFIα as the reference gene.

### 4.6. Statistical Analysis

The data were analyzed by one-way ANOVA and Duncan’s test using SPSS Statistics 20.0 (SPSS Inc., Chicago, IL, USA). The effects were considered significant if *p* < 0.05. GraphPad Prism 9.1.2 software was used to generate the figures.

## 5. Conclusions

In summary, the findings of this study suggest that exogenous MT improves drought tolerance in garden *chrysanthemum* seedlings by regulating their physiology and gene expression under water deficit conditions. The application of 100 μM exogenous MT increased the growth of the plants and improved the activity of the antioxidant system to alleviate the damage caused by drought stress and promote photosynthesis, and the expression of a series of downstream TFs, such as *TIFY, AP2/ERF-ERF*, *WRKY*, *GOX*, *MYB*, and *EIN*, was also induced by this treatment. Thus, exogenously applied MT could alleviate drought stress by reducing membrane lipid peroxidation, enhancing the antioxidant system, improving photosynthesis, and activating TFs and synthetic pathways. These results provide new insights into a probable mechanism through which MT induces protection against photosynthesis and enhances drought tolerance in *chrysanthemum* plants. In addition, they offer information on the mechanism of MT-mediated drought tolerance in *chrysanthemum*.

## Data Availability

The raw data underlying these analyses are available from the corresponding author upon reasonable request.

## Supplementary Materials

The following supporting information can be downloaded at https://www.mdpi.com/article/10.3390/plants12071489/s1, Table S1: CK_vs_MT.GO.classification and GO.enrichment; Table S2: KEGG-pathway_enrichment; Table S3: Primers used in this study.
