# Supplementary material for: Transcriptomic and Physiological Analyses Reveal the Molecular Mechanism through Which Exogenous Melatonin Increases Drought Stress Tolerance in Chrysanthemum
Source: Plants (Basel). 2023 Mar 29;12(7):1489. doi: 10.3390/plants12071489 (PMC10096800; doi:10.3390/plants12071489)
Supplement: Supplementary file 1 [file plants-12-01489-s001.zip › Supplementary Table S3 Primers used in this study.pdf]

Quantitative primer design

Supplementary Table S1 Quantitative primers of candidate genes

Gene name	Primer	Sequence(5’-3’)
EFIα	Forward primer	TTTTGGTATCTGGTCCTGGAG
	Reverse primer	CCATTCAAGCGACAGACTCA
AP2/ERF-AP2	Forward primer	AACTGTCCGTATCGTGAGAATG
	Reverse primer	CCAACTCATCCTCCTCATCATC
bHLH130	Forward primer	TCTGGGAAATCAACCACCTATG
	Reverse primer	ACGAATCTTACACGGAACAGAA
Tify9	Forward primer	GCTTTCGGATCGTCGAGATATT
	Reverse primer	GAAACCCGAGCTAGCAGATG
Tify6B	Forward primer	GCGGTATGGTGAATGTCTATGA
	Reverse primer	GTGTCCTTGGTTGAGGTGAA
Tify10A	Forward primer	CTTGGAGAAGAGGAAAGACAGAA
	Reverse primer	CCAAGTCCCAACCATGTCTTA
MYB62	Forward primer	CCTTACCATCCACAAACCAAAC
	Reverse primer	CGACAACATCTTCCCACTAGAA
MYBelated	Forward primer	GGACACCAAGTGACTCATGTATAG
	Reverse primer	ACGGATGTTGGGTTGAGTTAG
WRKY24	Forward primer	GCTGTATCACCCTCTTCGTATTT
	Reverse primer	CGGAGACGGCAATATGTTAGAA
EIN3	Forward primer	TGGGTTTCTTGATCGGTCTTC
	Reverse primer	GAGGTGGGTTCAGGCATTATAG
zf-HD	Forward primer	TCTGCTGACGGTTTGTCTTTA
	Reverse primer	CCGGTACTAGGTCTATGGTAGT
JOX1	Forward primer	AAAGCATTTGGTGGTGAAGATG
	Reverse primer	GGATCCGAGTGAGACGATAGA
GPX4	Forward primer	GGTTCCTTGGCGACAGTATTA

	Reverse primer	ACTTGTGGTGGGTGCATATC
HIP39	Forward primer	GGTGGTACTCAAGGTTCTTACC
	Reverse primer	CCCGATCACAGTTAGTTTCTGA
ABA4	Forward primer	CTCGTGGACACCTGATACAATAA
	Reverse primer	TGTATCCAAGCTGAGGCTAATG
GRAS	Forward primer	CACTAACTGCTGAAGGGCTAATA
	Reverse primer	AGAAGATAACCGAGCCACAAG
